# MIP-Modified Porous Silicon Optical Sensor for Interleukin-6 Label-Free Quantification

**DOI:** 10.3390/bios15050320

**Published:** 2025-05-17

**Authors:** Valeria Nocerino, Giulia Siciliano, Monica Bianco, Ilaria Rea, Principia Dardano, Maria Serena Chiriacò, Francesco Ferrara, Giuseppe Gigli, Elisabetta Primiceri, Luca De Stefano

**Affiliations:** 1 Institute of Applied Sciences and Intelligent Systems (ISASI), National Research Council (CNR), Via Pietro Castellino 111, 80131 Napoli, Italy; valeria.nocerino@na.isasi.cnr.it (V.N.); ilaria.rea@cnr.it (I.R.); principia.dardano@cnr.it (P.D.); 2 Department of Electrical Engineering and Information Technology, University of Naples Federico II, Via Claudio 21, 80125 Napoli, Italy; 3 Institute of Nanotechnology (Nanotec), Via Per Monteroni, 73100 Lecce, Italymonica.bianco@cnr.it (M.B.); mariaserena.chiriaco@nanotec.cnr.it (M.S.C.); francesco.ferrara@nanotec.cnr.it (F.F.);

**Keywords:** MIP, porous silicon, optical sensors, Interleukin-6

## Abstract

In this study, we present an innovative optical biosensor designed to detect Interleukin-6 (IL-6), a pivotal cytokine implicated in many pathological conditions. Our sensing platform is made of a porous silicon (PSi) nanostructured substrate modified with a thin (~5 nm) molecularly imprinted polymer (MIP), ensuring both high specificity and sensitivity toward IL-6 molecules. The fabrication process involves electrochemical etching of silicon chips to create the porous structure, followed by the electrodeposition of the MIP, which is tailored to selectively bind the IL-6 target. Extensive testing over a broad IL-6 concentration range demonstrates a clear, proportional optical response, yielding a limit of detection (LOD) of 13 nM. Moreover, the biosensor robustness was verified by evaluating its performance in bovine serum, a complex biological matrix. Despite the presence of various interfering components, the sensor maintained its selectivity and displayed minimal matrix effects, underlining its practical applicability in real-world diagnostic scenarios.

## 1. Introduction

The design and realization of biosensors, devices that convert a molecular interaction between a bioprobe and a target analyte into an easily processed electrical or optical signal by a transducer element, require two fundamental steps. The first is the proper passivation of the transducer surface for bioconjugating the bioprobe on it. This step also stabilizes the transducer surface against chemical modification during the measurement process, which could happen in the solution or after several washing passages. The second is the optimization of the transduction mechanism, which allows the generation of a useful signal even at very small analyte concentrations. In this view, porous materials, offering a huge specific surface area, where many bioprobes can be allocated, are natural candidates for hosting bioprobes and revealing the interaction with their target analytes. In the last few years, many works have reported the use of porous silicon (PSi) in chemical and biological sensing, ranging from healthcare diagnostics to environmental monitoring [1,2]. The unique characteristics of PSi, including air-filled, tuneable size pores, custom chemically modifiable large surface area [3] that can host a huge number of bioprobes, together with biocompatibility, photoluminescence, and biodegradability, make it an appealing optical transducer in biosensing systems [4,5,6]. The PSi reflectivity (and transmissivity) spectrum can show evident sharp optical resonances, as it happens in photonic crystals, or typical fringe patterns in periodic structures such as Bragg mirrors and rugate filters [7]. PSi optical sensors monitor the changes of photoluminescence or reflectivity spectra when something penetrates its sponge-like matrix, due to the substitution of part of the air into the PSi pores that changes the optical density of the PSi structure [8,9,10]. Even if the mechanism is specific, since it is ruled by the chemical and physical properties of each analyte, PSi itself is not selective as a sensor, i.e., it can be used just for the recognition of pure substances, not for quantifying the amount of an element in a complex mixture [11]. In this sense, the performance of PSi-based sensors can be enhanced by modifying the PSi surface by exploiting the natural selectivity of the bioprobes that can be complexed with the target analytes [12,13,14,15]. In the biosensor design, molecular recognition is thus a crucial issue, and the choice of recognition element influences the selectivity of the analytical system and even the sensitivity.

The most common approach is based on natural or synthetic biomolecules such as antibodies, nucleic acids, or aptamers [16]. However, the use of these recognition elements has several drawbacks, such as high cost and low stability [17]. An alternative approach is replacing natural or synthetic bioprobes with biomimetic receptors that can offer improved stability, cost-effectiveness, and means of rapid fabrication [18].

Among the different available strategies, the use of polymers for PSi surface modification has emerged as an innovative tool in biosensing due to their unique properties, including chemical stability, as well as electronic and mechanical properties [19,20,21]. Thus, integrating polymers and PSi devices is a valuable strategy to improve selectivity and sensitivity and to add new functionalities for molecular recognition [22,23,24].

Amid the huge family of available polymers, molecularly imprinted polymers (MIPs) are synthetic receptors utilized as mimetic antibodies for selective molecular recognition, which provide templates able to non-covalently bind their antigens with the corresponding (imprinted) molecular morphology [25]. Indeed, the use of MIPs can both stabilize the PSi surface against corrosion in basic solutions and add remarkable selectivity and affinity, making the hybrid organic–inorganic system an ideal candidate for specific target detection. Several works have reported the use of MIP based on *o*-phenylenediamine (*o*-PD) electro-polymerization for detecting various target molecules [26,27,28].

Cytokines are signaling proteins produced by various cells in the immune system. They are often used as biomarkers for disease monitoring, such as cancer progression [29], hepatic inflammation [30], and liver diseases. In particular, Interleukin-6 (IL-6) is a prototypical pleiotropic cytokine polypeptide secreted into the serum by T cells and macrophages, which plays an important role in many bodily processes, such as chronic inflammation, acute phase response, autoimmunity, and fibrogenesis [31]. During infections or after tissue injuries, IL-6 is promptly produced by monocytes and macrophages and contributes to removing infectious agents and restoring damaged tissues by activating immune, hematological, and acute-phase responses. Once the stress in the host is over, the synthesis of IL-6 ends, so uncontrolled, excessive, or persistent IL-6 production is related to a pathological role in the development of various inflammatory diseases and cancers [32].

Therefore, IL-6 can be considered a useful biomarker in different kinds of diseases, such as cardiovascular diseases [33], cancer [34], lung fibrosis [35], and chronic intestinal inflammation [36]. Nowadays, the detection of IL-6 is mostly based on ELISA immunoassays and chemiluminescent immunoassays that exploit enzyme-linked immunoassays for their quantification with high sensitivity (≈10–20 pg/mL), specificity, and reliability [37]. Despite several benefits of these techniques, they are costly, time-consuming, and must be performed by skilled personnel. On the other hand, point-of-care devices (POC) are essential for detecting IL-6-related diseases such as sepsis, where diagnosis within the first 6 h of disease onset and the administration of specific therapy could significantly improve patient outcomes [38]. For this reason, the development of selective, rapid, and cost-effective sensing techniques, together with the possibility of miniaturization and portability for the detection of IL-6, is crucial for early screening. In this work, we combined the properties of PSi with MIP technology to develop an optical PSi transducer modified with a thin layer of poly (o-PD) (P*o*-PD), envisioned as a proof-of-concept sensor for point-of-care, liquid-biopsy detection of the cytokine IL-6. The artificial IL-6 receptor was synthesized by electro-polymerizing the functional monomer *o*-phenylenediamine (o-PD) in the presence of IL-6, directly inside the PSi matrix. A preliminary version of this platform, limited to the fabrication of the porous-silicon scaffold and P*o*-PD imprinting, was presented in our previous work [39]. The present study moves beyond that initial demonstration by adding complete quantitative calibration, rigorous selectivity assays, and validation in complex biological matrices. In particular, after the template-removal procedure, rebinding assays were performed, and the analytical performances of the developed sensor were evaluated in buffer solution and a complex matrix. The selectivity of the synthetic transducer was tested against several interfering molecules. The results demonstrated high affinity and specificity for IL-6, confirming the sensor’s capability to distinguish it from structurally similar analytes. Overall, the sensor performance highlights the potential of this approach for selective and sensitive IL-6 detection in liquid biopsy at the point-of-care. The method offers rapid and straightforward analysis, making it a promising tool for real-time cytokine monitoring in clinical applications.

## 2. Materials and Methods

### 2.1. Chemicals

*O-phenylenediamine* (*o*-PD, ≥98%, Sigma-Aldrich, Merck KGaA, Darmstadt, Germany), Interleukin-6 (powder, Sigma-Aldrich), Phosphate Buffered Saline (PBS ≥ 98%, Sigma-Aldrich), Acetate Buffer (AC ≥ 98%, Sigma-Aldrich), Hydrofluoric acid (HF), Absolute ethanol (EtOH), Isopropanol (IPA), Peroxidase (HRP, Sigma-Aldrich), Ascorbic Acid (AA, Sigma-Aldrich). L-cysteine (L-Cys, Sigma-Aldrich). All solvents, purchased from Sigma-Aldrich, are of the highest purity available. All aqueous solutions were prepared by using water obtained from a Milli-Q Gradient A-10 system (Millipore, Merck KGaA, Darmstadt, Germany, 18.2 MΩ cm, organic carbon content ≤ 4 µg/L).

### 2.2. Preparation of IL-6 and o-PD Solution

A stock solution of IL-6 (100 µg/mL) was prepared in phosphate buffer solution (PBS) at pH 7.4 and stored at −20 °C if not used. A standard stock solution of *o*-PD (0.1 mg/mL) was prepared in an acetate buffer solution (pH 5.2) [27].

### 2.3. Porous Silicon Fabrication

A PSi single-layer structure was realized by electrochemically etching n-type crystalline silicon with a resistivity of 0.01–0.02 Ω cm, oriented in the <100> direction and having a thickness of 500 µm. The etching was carried out in 200 mL of hydrofluoric acid (5% by weight) and ethanol at room temperature by applying a current density of 20 mA cm^−2^ for 90 s. Before etching, the native oxide layer on the silicon substrate was removed by immersing it in 10 mL of hydrofluoric acid (1% by weight) and ethanol for five minutes, and then rinsed with air. The resulting PSi monolayer exhibited a porosity of 61% (with a refractive index of 1.83 at a wavelength of 1.2 µm), a thickness of 2.1 µm, and a pore size distribution ranging from 50 to 250 nm. Small drifts in current density or HF concentration can shift porosity by ± 2–3%, local heating during prolonged etching may broaden the pore-size distribution, and wafer-to-wafer variability can affect film thickness and optical reproducibility. To mitigate these issues, the electrolyte was prepared immediately before use and maintained at 20 °C, and the current source was calibrated before every run. These precautions limited the relative standard deviation of the effective optical thickness across five independently fabricated wafers, yielding a consistent porosity and pore-size range of 50–250 nm.

### 2.4. Reflectance Spectroscopy

The optical setup used to obtain the reflectivity spectra of the PSi monolayer consisted of a white light source and an Ando AQ6315B optical spectrum analyzer (Yokohama Italia S.r.l., Nova Milanese, Italy). A Y optical fiber reflection probe (Avantes, Apeldoorn, The Netherlands) illuminated the porous silicon sample and collected light at normal incidence. The acquired optical spectra were recorded from 600 to 1600 nm, with a sampling step of 1 nm. Each reflectivity spectrum reported and utilized for further elaborations was averaged from three independent measurements.

### 2.5. Electrochemical Measurements

Electrochemical measurements were performed using an Autolab potentiostat (PGSTAT 204, Metrohm) in a standard three-electrode configuration. The n-doped porous silicon device and a platinum wire were used as working and counter electrodes, respectively, while an Ag/AgCl/KCl (3M KCl) electrode was used as a reference. All measurements were performed at room temperature (22 °C).

### 2.6. IL-6 MIP Synthesis, Template Removal, and Rebinding

Poly (ortho-phenylenediamine) (P*o*-PD) film was synthesized by exploiting the experimental procedure detailed in [27]. In particular, P*o*-PD was directly electrosynthesized on PSi substrate (acting as a working electrode) by applying cyclic voltammetry (CV) (5 scans) in the potential range −0.2–0.8 V vs. Ag/AgCl at a scan rate of 50 mV/s in a solution of acetate buffer (0.5 M, pH 5.2) containing 0.1 mg/mL *o*-PD. Before polymerization, IL-6 was added to the *o*-PD solution as a template molecule at a 100 µg/mL concentration. After polymerization, the modified PSi sensor was washed with a solution of acetic acid 5% in water to remove the template. The binding properties and sensing performance of the MIP-modified sensors were then evaluated by incubating them with various concentrations of IL-6 in PBS for 1h. After incubation, the sensors were rinsed with PBS to eliminate any non-specifically adsorbed proteins. As a negative control, a non-imprinted polymer (NIP) was synthesized following the same procedure, but without IL-6 as a template. Rebinding experiments were also conducted in bovine serum diluted to 50% in PBS to assess sensor performance in a complex biological matrix and evaluate potential interference effects.

### 2.7. Atomic Force Microscopy

PSi samples, before and after MIP modification, were visualized by an XE-100 atomic force microscope (Park Systems Europe GmbH, Mannheim, Germany). The samples’ surfaces were imaged in Non-Contact Mode (NCM), using silicon/aluminum coated cantilevers (SSS-NCHR 10M; Park Systems Europe GmbH) 125 μm long with a resonance frequency of 204 to 397 kHz, nominal force constant of 42 N m^−1^, and a typical tip radius 2 nm (<5 nm max). The scan frequency was typically 0.35 Hz per line. AFM images were elaborated by the XEI 1.8.1.build214 program (Park Systems Europe GmbH).

### 2.8. Spectroscopic Ellipsometry

Ellipsometric measurements of MIP deposited on silicon were conducted using a Jobin Yvon UVISEL-NIR phase-modulated spectroscopic ellipsometer apparatus. The analysis was performed at the incidence angles 60°, 65°, and 70°, over the spectral range 300–1700 nm, with a resolution of 10 nm. The thickness and optical constants (refractive index *n* and extinction coefficient *k*) of the films deposited on silicon were determined from the analysis of ellipsometric data using DELTA PSI software (Horiba Jobin Yvon. DELTA PSI Software manual, ver.2.4.3 158).

### 2.9. QCM-D Studies

A Q-Sense E1 system (Q-sense, Sweden) was used to monitor the template-removal procedure in real time by detecting the mass changes following the IL-6 release from the MIP-modified QCM crystal surface. As a negative control, a QCM-D study on NIP-modified QCM crystal was performed. A gold-coated 5 MHz, 14 mm diameter, AT-cut quartz crystals (QSX 301, Q-Sense, Sweden) were used as a substrate for MIP and NIP synthesis. Before each measurement, QCM crystals were pretreated according to routine cleaning protocols [40]. Simultaneously, the resonance frequency and energy dissipation were measured at the fundamental harmonics (n = 1 at 5 MHz) and six overtones to the fundamental (n = 3, 5, 7, 9, 11, 13 at 15 MHz, 25MHz, 35MHz, 45MHz, 55MHz, 65 MHz, respectively). For simplicity, only changes in the frequency, mass, and dissipation of the ninth overtone (45 MHz) were presented in the reported sensorgrams. In our work, the measured dissipation signals remain relatively low, then the mass density measured (mass per unit of the crystal area), referred to as Δm, is proportional to frequency changes, referred to as Δf, according to the Sauerbrey [41]:

Δm = −C × Δf/n

where *n* is the considered overtone number and C is a constant approximately equal to 17.7 ng/(cm^2^·Hz) for the 5 MHz AT-cut quartz crystal at room temperature. It is well known that this equation can be applied with high accuracy to rigid films with no meaningful changes in dissipation values [42].

### 2.10. Contact Angle Measurements

The sessile drop method was used to measure static water contact angles at room temperature (22 °C ± 1 °C) with a CAM 200 (KSV Instruments Ltd., Finland) instrument. The reported data are the average of three measurements on different areas of the bare and MIP or NIP-modified QCM gold crystal surfaces before and after QCM measurements to evaluate the wettability effect of the removal of acidic treatment during QCM tests. The errors were calculated as the standard deviations.

## 3. Results and Discussion

### 3.1. Synthesis and Characterization of the IL-6 MIP Receptor on Porous Silicon

**Figure 1:**
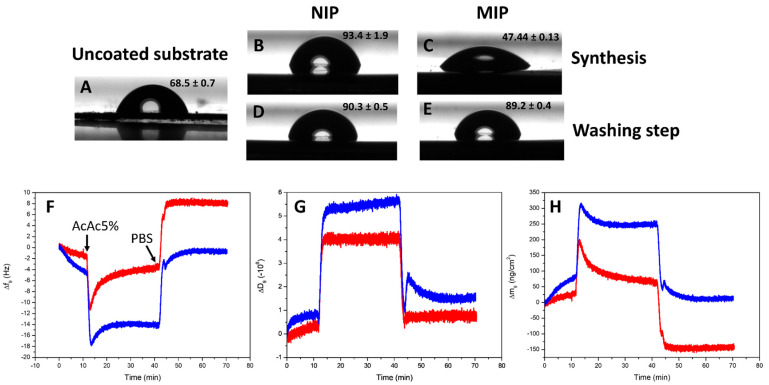
(**A**–**E**) Contact angle measurements acquired at room temperature (22 ± 1 °C). Water droplet profiles on QCM-D gold-coated surfaces: (**A**) unmodified surface, (**B**) surface coated with non-imprinted polymer (NIP), (**C**) surface coated with molecularly imprinted polymer (MIP), (**D**) NIP surface after QCM negative test with 5% acetic acid, and (**E**) MIP surface after QCM template-removal test using 5% acetic acid. (**F**–**H**) QCM-D analysis at the ninth overtone, performed at room temperature (22 ± 1 °C). Real-time responses of MIP-coated (red curves) and NIP-coated (blue curves) gold sensors upon exposure to 5% acetic acid solution (flow rate: 100 μL/min), followed by a final PBS rinse at the same flow rate. Panels show (**F**) frequency shift (Δf), (**G**) dissipation change (ΔD), and (**H**) mass change.

Before the modification of the PSi film by the imprinted polymer, the IL-6 MIP film deposited on the flat gold surface of the QCM crystal was deeply investigated by water contact angle and quantitative QCM-D measurements. After the electropolymerization of the IL-6 MIP film on the QCM support (both the MIP and NIP layers were directly polymerized onto the QCM sensor surface), a dedicated washing step was optimized to effectively remove the template molecule entrapped in the MIP matrix. In our previous studies, alkaline solutions based on NaOH were successfully employed for this purpose. However, in the present case, such basic conditions could compromise the integrity of the porous silicon substrate. To overcome this limitation, a milder acidic treatment using 5% acetic acid was tested. The contact angle measurements were performed to monitor changes in surface wettability that strongly depended on its composition. The water contact angle data were analyzed on the QCM gold crystal before (Figure 1A–C) and after (Figure 1D,E) the acidic treatment in real time during QCM-D tests. The uncoated substrate exhibited a contact angle of ~68° (Figure 1A), which decreased to ~47° after deposition of the MIP (Figure 1C), suggesting increased hydrophilicity likely due to the presence of the proteins mostly on the MIP surface. The NIP showed a much higher contact angle (~93°), consistent with the absence of the protein template (Figure 1B). After the washing step by a 5% acetic acid solution, the NIP surface remained nearly unchanged (~90°) as expected, since there was nothing to remove, while the MIP surface underwent a marked shift to ~89°, aligning closely with the NIP value (Figure 1D,E). This result indicated that the hydrophilic character of the MIP was primarily conferred by the embedded IL-6 template, and also its almost complete removal after the washing step, restoring the intrinsic wettability of the polymer. In addition, QCM analysis provided quantitative confirmation of the template removal. Once the MIP or NIP-modified crystals were inserted in the QCM-D chamber, the flow cell was filled with PBS at 100 mL/min, and after 10 min equilibration, a 5% acetic acid solution was injected for 30 min. Frequency (∆f) and dissipation (∆D) shifts were recorded in real time during all QCM experiments. At the end of acid-removal washing, a final PBS washing was performed at 100 mL/min. After these two washing steps, a significant desorption signal was recorded for the MIP (∼150 ng/cm^2^), while no appreciable mass change was observed for the NIP. This differential response confirmed that the observed mass loss in the MIP was due to the selective removal of the IL-6 template molecule, rather than to nonspecific dissolution or degradation of the polymer matrix (Figure 1F–H). Based on the observed mass loss and the molecular weight of the template (27 kDa), the amount of desorbed material corresponded to approximately 5.56 × 10^−12^ mol/cm^2^, or 3.35 × 10^12^ molecules/cm^2^. These results provided quantitative evidence of successful template entrapment during MIP formation and its subsequent selective removal, a critical prerequisite for the generation of specific recognition sites in the polymer network.

**Figure 2:**
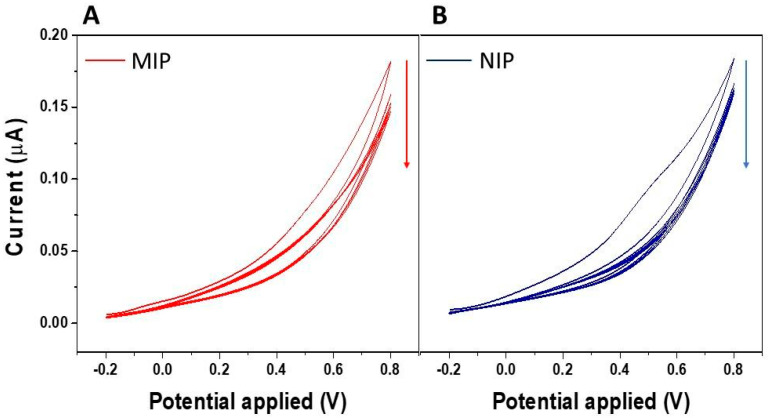
Electrochemical deposition of P*o*-PD film in the presence of IL-6 as a template (**A**) and its absence (**B**) by cyclic voltammetry.

A single-layer PSi structure was fabricated to act as the optical transducing platform. The PSi was made by electrochemically etching an n-type crystalline silicon wafer. The resulting PSi monolayer exhibited a porosity of approximately 61%, a thickness of 2.1 µm, and a pore size distribution ranging from 50 to 250 nm [43]. The 61% porosity and the 50–250 nm pore-size range supply a large internal surface area for imprinting while creating channels wide enough for the IL-6 molecule to diffuse rapidly in and out of the polymer matrix. At the same time, the 2.1 µm film thickness preserves the interferometric fringe pattern required for optical read-out. MIP-film was synthesized by cyclic voltammetric deposition (5 scans) of o-PD in the presence of IL-6 into PSi devices (Figure 2A). Figure 2 reports a typical cyclic voltammogram recorded during the electro-polymerization of o-PD in the presence of IL-6. A significant decrease in the anodic peak was observed from the 1st to the 5th cycle, indicating irreversible monomer oxidation on the electrode surface during continuous cycling and demonstrating the formation of a non-conductive polymer film on the electrode surface. Similar trends were observed in the cyclic voltammogram evolution both in the presence of IL-6 (MIP, Figure 2A) and its absence (NIP, Figure 2B), indicating that only the monomer underwent electro-polymerization.

**Figure 3:**
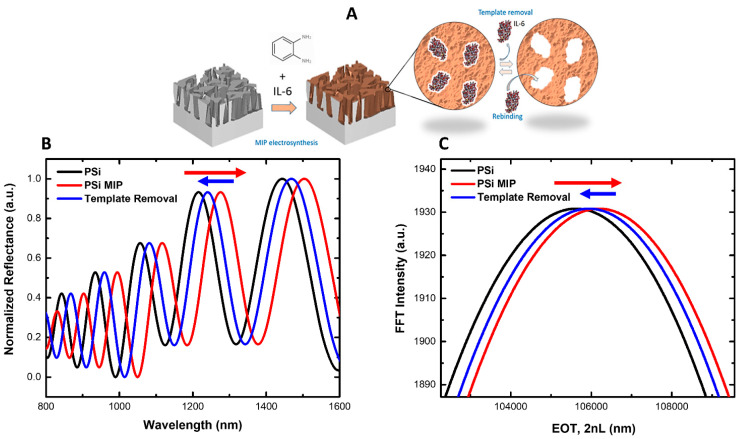
(**A**) Schematic representation of the MIP-modified PSi fabrication process. (**B**) Reflectance spectra of bare PSi (black line), MIP-modified PSi (red line), MIP-modified PSi after template removal (blue line), and (**C**) their respective FFT analysis.

After this preliminary characterization, the system was characterized using optical measurements at three critical stages: after PSi fabrication via electrochemical etching, immediately after MIP deposition, and following template (IL-6) removal using a 5% acetic acid solution in water. Reflectance spectra were recorded at each stage to assess structural and refractive index variations, allowing real-time monitoring of shifts in the photonic resonance of the PSi layer. These spectral shifts indicate changes in the effective refractive index within the porous structure (Figure 3B). A clear redshift of 60 nm in the reflectance peak was observed after electropolymerization, confirming the successful deposition of the MIP layer compared to the bare PSi substrate. This shift can be attributed to the infiltration of the non-conductive polymer matrix into the porous network, effectively increasing the overall refractive index [2,43]. After the template removal in acetic acid 5%, a noticeable blue shift (30 nm) in the reflectance peak was detected. The removal of the template protein from the polymer cavities reduces the mass within the pores and thus lowers the local refractive index. Consequently, the reflectance spectra shift back to shorter wavelengths, proving that IL-6 had been successfully removed, thereby exposing the imprinted binding sites. Finally, the reflectance spectra were also analyzed using Fourier Transform analysis, which confirmed the redshift after MIP electrodeposition and the blue shift after the template removal (Figure 3C).

**Figure 4:**
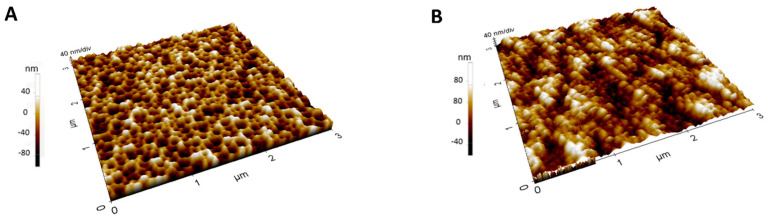
(**A**) AFM characterization of bare PSi, and (**B**) MIP-modified PSi.

It is important to note that following the 60 nm red shift observed after MIP electropolymerization, a 30 nm blue shift was recorded upon template removal, reflecting the extraction of IL-6 and the resulting decrease in refractive index within the PSi layer. It is worth noting that the polymer electrosynthesis process involved the use of 100 µg/mL of *o*-phenylenediamine (o-PD) and the addition of 100 µL of IL-6 at a concentration of 100 µg/mL, ensuring that the PSi matrix was partially occupied by both the polymer and the target template. Consequently, upon the template-removal step, the extraction of IL-6 from the MIP structure reduced the overall refractive index within the porous layer, leading to the observed blue shift. This outcome aligns with the expected behavior of the MIP-modified PSi system, wherein target removal manifests as a decrease in refractive index and a corresponding shift in the reflectance spectrum. The surface topography of PSi, both before and after MIP functionalization, was examined using AFM, as shown in Figure 3. In Figure 4A, the AFM images of the bare PSi (without MIP) exhibit characteristic hillocks and voids, with the voids (dark regions) measuring around 100 nm in diameter and uniformly distributed across the substrate. These features confirm the intrinsic porous nature of the PSi, providing a high surface area that is potentially advantageous for subsequent biomolecular recognition. In contrast, Figure 4B demonstrates the morphological impact of MIP functionalization.

**Figure 5:**
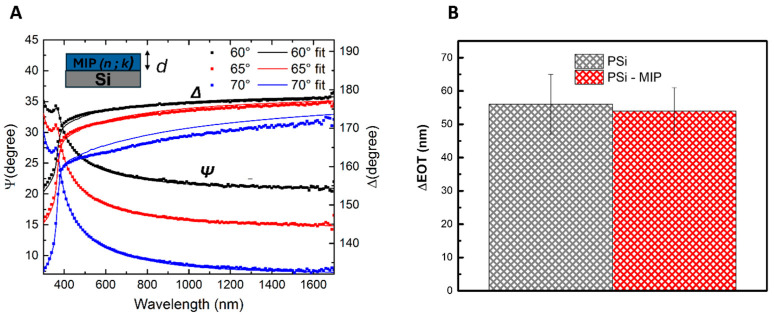
(**A**) Measured (dots) and calculated (solid lines) Y and D spectra of MIP deposited on a silicon substrate. Spectra were acquired at the incidence angles 60°, 65°, and 70°. In the inset ellipsometric model of MIP deposited on silicon, measured (dots) and calculated (solid lines) Y and D spectra of MIP deposited on a silicon substrate. Spectra were acquired at the incidence angles 60°, 65°, and 70°. (**B**) ΔEOT analysis following the IPA filling test on bare PSi and MIP-modified PSi. The optical measurements are obtained from a minimum of three independent samples (*n* ≥ 3). SDs are reported as vertical bars.

The partial blocking of pores observed here is attributed to the deposition of the MIP layer, visible as larger, lighter-toned polymer aggregates that diminish the visibility of the underlying porous matrix. To estimate the thickness of the MIP film, ellipsometric measurements of MIP deposited on silicon were conducted. The analysis was performed at the incidence angles 60°, 65°, and 70°, over the spectral range 300–1700 nm, with a resolution of 10 nm [44]. The instrument measured the spectral variation of the ellipsometric angles Ψ and Δ, defined by the following relationship:

$${tg\Psi e}^{i∆}=\frac{Rp}{Rs}$$

where *Rp* and *Rs* represent the complex reflection coefficients of the light polarized parallel and perpendicular to the plane of incidence, respectively. The results of this analysis were obtained as an average of three separate measurements on the same sample, allowing for a statistical consideration of film inhomogeneities. MIP films were modeled assuming that the real and imaginary parts of the refractive index are non-dispersive in the measured spectrum, i.e., *n* (*λ*) = const and *k* (*λ*) = const, where *λ* is the wavelength. Figure 5A shows the spectra of the measured and calculated ellipsometric angles Y and D of MIP deposited on silicon at incidence angles 60°, 65°, and 70°. The fitting procedure yielded a refractive index *n* = 1.63 ± 0.01 and a thickness *d* = 4.18 ± 0.03 nm (χ^2^ = 0.49). Following the AFM-based morphological characterization and the ellipsometric analysis, which confirmed the presence of a very thin film of MIP on the PSi surface, a filling test with isopropanol (IPA) was conducted to verify that the pores remained accessible and were not completely occluded by the polymer layer. Maintaining partially open pores is crucial because, while the MIP must cover the PSi to create imprinted sites, the sensing mechanism relies on the target analyte (in this case, IL-6) being able to diffuse into those imprinted cavities. Bare PSi and PSi functionalized with MIP were immersed in IPA for 5 min, and the reflectance spectra were recorded before and after immersion. IPA was chosen for the filling test due to its low surface tension and viscosity, which facilitate deep infiltration into the porous silicon layer without forming significant air pockets. Additionally, IPA is minimally reactive with both the PSi framework and the thin MIP film, ensuring the polymer remains intact during testing. The relatively rapid evaporation further simplifies post-immersion measurements. To quantify infiltration, the shifts in the Fast Fourier Transform (FFT) of the reflectance spectra were measured for both sample types before and after isopropanol immersion and then compared. Data revealed that both the bare and MIP-modified PSi exhibited comparable shifts in their FFT (55 nm) peaks following IPA infiltration (Figure 5B).

This equivalence indicates that, despite the presence of the MIP layer, the pores remained largely accessible to the solvent, confirming that the polymer did not occlude the pore network. The thin nature of the deposited MIP thus provided adequate surface coverage to create imprinted sites while still allowing IPA—and by extension, potential analytes—to diffuse through the PSi layer. Hence, the filling test demonstrated the optimal balance between effective MIP functionalization and porosity available.

### 3.2. MIP-PSi Sensor Calibration and Testing

**Figure 6:**
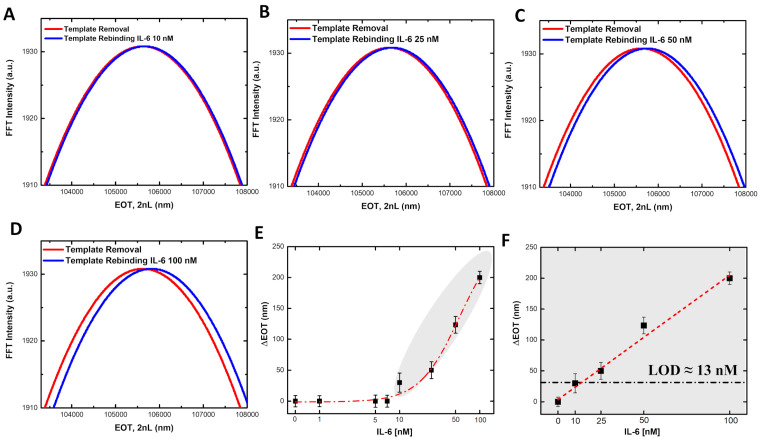
(**A**–**D**) FFT analysis of reflectance spectra after the template removal (red line) and after the template rebinding with different concentrations of IL-6 (blue line). (**E**) Calibration curve of IL-6 in standard solution, on x-axis IL-6 concentrations in [nM], on y-axis ΔEOT values calculated as EOT_IL-6_ − EOT_blank_. (**F**) Linear Range of biosensor. The gray area in panels E and F indicates the linear dynamic range of the biosensor, where the optical response shows a direct proportionality to IL-6 concentration. The optical measurements are obtained from a minimum of three independent samples (*n* ≥ 3). SDs are reported as vertical bars.

After completing and verifying each step of the PSi functionalization, the MIP-modified PSi platform was employed for IL-6 detection. Specifically, the sensor was incubated with a series of IL-6 solutions at increasing concentrations (1 nM, 5 nM, 7 nM, 10 nM, 25 nM, 50 nM, 100 nM). The MIP-modified PSi sensor was incubated with 200 µL of IL-6 in PBS for 1 h, allowing interaction between the target protein and the imprinted cavities. After the incubation period, the sample was washed three times, twice with PBS and once with deionized water, to remove any unbound IL-6 molecules. The sensor was then analyzed for shifts in its reflectance spectra. The corresponding FFT analysis of the reflectance data for all concentrations that produced a detectable redshift (indicating IL-6 binding) is also presented (Figure 6A–D). As demonstrated in Figure 6F (gray area), for each IL-6 concentration, the change in effective optical thickness (ΔEOT = EOT*_IL_*_-6_ − EOT*_Blank_*), calculated from the FFT, is directly proportional to the concentration of IL-6. This trend highlights the ability of our sensor to produce measurable and consistent reflectance signal variations across a range of IL-6 concentrations, confirming the principle of selective interaction and the effectiveness of the MIP-modified PSi platform for IL-6 detection. A calibration curve was obtained as a function of IL-6 concentrations in the range from 10 nM to 100 nM, with the linear regression described by the equation y = 2.2 (±0.2) [nM]^−1^ x, with an R^2^ = 0.99 (Figure 6F). A LOD of 13 nM was obtained (3 × standard deviation/sensitivity, 3σ_blank_/S [45,46].

The QCM characterization of the IL-6 MIP film before and after the washing steps allowed estimating the maximum effective analyte concentration that can be detected by the sensor under diffusion-limited conditions. Assuming a diffusion layer thickness of 100 µm, the MIP surface binding capacity of 5.56 × 10^−12^ mol/cm^2^ and a sensing area of 1 cm^2^, the total amount of analyte that could be captured at saturation conditions was 5.56 × 10^−12^ mol. The corresponding diffusion volume was approximately 10 µL, resulting in a theoretical maximum effective concentration of ~0.556 µM (556 nM). This rough estimation was in good agreement with our experimental data, which showed a linear calibration curve up to 100 nM without signs of saturation.

**Figure 7:**
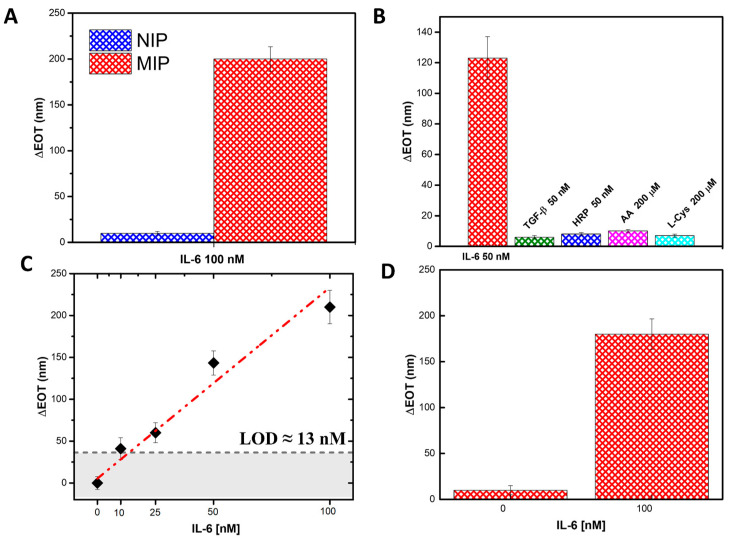
(**A**) Histograms comparing the ΔEOT values for MIP-modified PSi incubated with IL-6 versus NIP-modified PSi incubated with the same concentration of IL-6. (**B**) Histograms comparing the ΔEOT values for MIP-modified PSi incubated with IL-6 and TGF-β, HRP, AA, and L-Cys as interfering molecules. (**C**) Calibration curve in serum solution. (**D**) Histograms comparing the ΔEOT values of MIP-modified PSi incubated with only Bovine Serum solution and Bovine Serum solution and IL-6. The optical measurements are obtained from a minimum of three independent samples (*n* ≥ 3). SDs are reported as vertical bars.

To evaluate the performance and specificity of our biosensor, the highest concentration of IL-6 (100 nM) was incubated with PSi modified with a non-imprinted polymer (NIP). Reflectance spectra were acquired, and the EOT values were calculated using FFT analysis. As shown in Figure 7A, no significant shift in the reflectance signal was observed for the NIP-functionalized PSi after incubation with IL-6, in contrast to the MIP-functionalized PSi, which exhibited a clear shift. This confirms that the NIP does not recognize or bind IL-6, further validating the specificity of the MIP-modified sensor.

Additionally, the specificity of the IL-6-imprinted PSi sensor was challenged with four common interferents: transforming growth factor-β (TGF-β, 50 nM), horseradish peroxidase (HRP, 50 nM), ascorbic acid (AA, 200 µM), and L-cysteine (L-Cys, 200 µM). The concentration range was chosen to exceed typical physiological levels to assess the selectivity. After 1 h of incubation, the IL-6-imprinted PSi sensor showed no significant shift in the reflectance spectra, demonstrating the absence of non-specific binding or fouling (Figure 7B). Under identical conditions, exposure to 50 nM IL-6 produced a distinct red shift in the reflectance spectra, confirming that the molecularly imprinted cavities recognize and bind the target cytokine selectively, even in the presence of structurally and chemically diverse species. Moreover, the specificity of the synthesized MIP was also tested by investigating the cross-reaction of the MIP with the interferent TGF-β. The obtained cross-reactivity value, calculated as the ratio between the interferent and MIP responses, showed a value of 20% for TGF-β, thus suggesting a poor interaction with the MIP film. These results confirm the high selectivity of the MIP-modified PSi biosensor for IL-6 detection. As a final evaluation of the biosensor performance, different concentrations of IL-6, from 10 nM to 100 nM, were tested in a complex matrix, specifically 50% bovine serum diluted in PBS. The results were analyzed using FFT, and a calibration curve was constructed by plotting the ΔEOT values as a function of IL-6 concentration. The obtained linear fitting is described by the equation y = 2.1 (±0.2) [nM]^−1^ x, with an R^2^ = 0.99 (Figure 7C). To assess the impact of the biological matrix, the ratio between the calibration curve slopes in standard solutions and the serum matrix was calculated, yielding a value of 1.03. This indicates that the serum matrix had a minimal effect (~0.03%), confirming the sensor’s robustness and reliability even in complex environments. A control experiment was also carried out to confirm the absence of matrix effects. Specifically, Figure 7D presents a histogram of the ΔEOT values obtained by incubating the MIP-modified PSi both in bovine serum alone and bovine serum containing IL-6. From the graph, it is evident that in the absence of IL-6, no significant shifts are observed, thereby confirming the negligible influence of the matrix under these conditions. This demonstrates that the sensor maintained excellent performance and sensitivity in a more complex biological environment, confirming its potential for real-world applications in IL-6 detection and future point-of-care diagnostic platforms.

Finally, the imprinting factor, defined as the ratio between MIP-modified PSi and NIP-modified PSi response recorded and calculated for a concentration of 100 nM, showed a high value equal to 21, demonstrating a strong increase in the interaction between IL-6 and the imprinted polymer compared to the non-imprinted one. These results confirm the superior ability of the MIP to specifically recognize and bind IL-6 compared to the NIP and further validate the efficiency of the MIP modification in ensuring a highly selective biosensor with minimal interference from non-specific binding [27].

**Figure 8:**
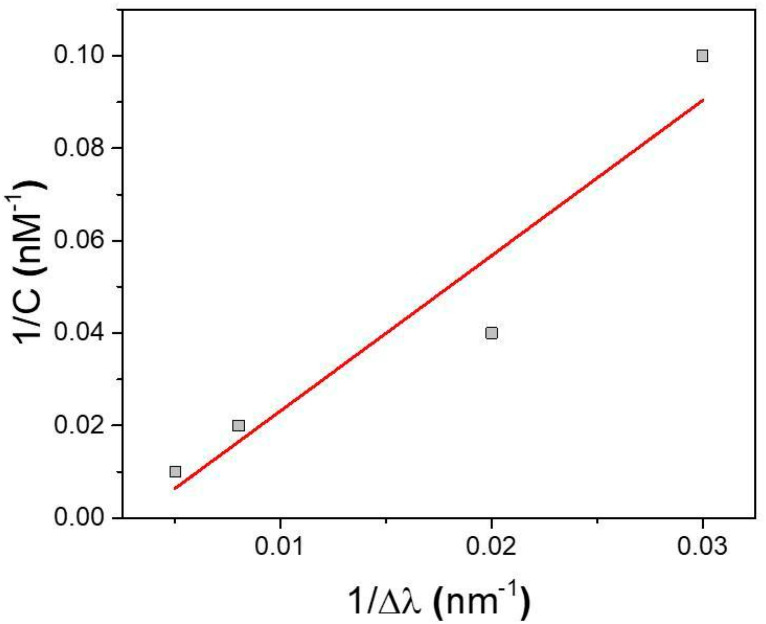
Scatchard plot for MIP-PSi sensor.

The dissociation constant K_D_, estimated by Scatchard plot analysis of binding data [47], reveals a remarkable value of 100 ± 25 nM for the MIP-PSi sensor, indicating a high affinity of the imprinting sites for IL-6. Details of the calculation are reported in Figure 8.

## 4. Conclusions

In this work, we developed and validated a MIP-modified porous silicon (PSi) biosensor for the detection of Interleukin-6 (IL-6), demonstrating high specificity, sensitivity, and robustness. By integrating the unique optical properties of PSi with the selectivity of molecularly imprinted polymers (MIPs), we successfully engineered a label-free sensing platform capable of recognizing IL-6 with excellent affinity. Our results confirmed that the sensor provides concentration-dependent optical shifts, enabling a quantitative detection of the analyte with a limit of detection (LOD) of 13 nM. This level of sensitivity is promising for early-stage cytokine monitoring, a critical factor in disease progression assessment and patient management. Additionally, the biosensor maintained its performance in complex biological matrices, exhibiting minimal matrix effects (~0.03%). The specificity of the sensor was further validated through control experiments using non-imprinted polymer (NIP) and interfering cytokines (TGF-β), showing negligible non-specific binding and demonstrating the high selectivity of the imprinting process. Additionally, the imprinting factor of 21 highlights the superior binding capacity of the MIP-functionalized PSi surface compared to the non-imprinted counterpart, suggesting a successful imprinting process. The dissociation constant (K_D_), estimated through Scatchard plot analysis, revealed a high-affinity interaction (K_D_ = 100 ± 25 nM), further supporting the efficiency of the molecular imprinting strategy. This MIP-PSi biosensor is label-free, eliminating the need for secondary reagents, enzymatic amplification, or nanostructured labels. This streamlines the assay, reducing time, cost, and potential sources of variability. Fabrication is rapid, reproducible, and readily scalable: a single electropolymerization step deposits o-phenylenediamine onto the porous-silicon substrate, and the resulting molecularly imprinted polymer, templated with IL-6 itself, serves as the selective recognition element. Many high-sensitivity biosensors rely on multi-step surface chemistries, nanoparticle labels, or complex amplification strategies that hinder practical deployment. A comparative summary table (Table S1, Supporting Information) summarizes recent IL-6 biosensors, highlighting linear range limit of detection, labeling strategy, and fabrication complexity [48,49,50,51,52]. While our device is not the most sensitive ever reported, it strikes a compelling balance between performance and simplicity, making it a strong candidate for point-of-care and on-chip applications. The advantages of the developed sensor lie in its robustness, rapid and straightforward detection process, scalability, and potential integration into point-of-care (POC) diagnostic platforms. Unlike conventional ELISA and chemiluminescent immunoassays, which require costly reagents, time-consuming procedures, and specialized personnel, such a biosensor offers a cost-effective and user-friendly alternative. In conclusion, the proposed MIP-PSi biosensor represents a promising and innovative tool for real-time IL-6 detection, with potential applications in clinical diagnostics, personalized medicine, and inflammatory disease monitoring. Future work will focus on further miniaturization, automation, and integration with microfluidic systems for enhanced portability and real-world applicability in liquid biopsy-based diagnostics.

## Data Availability

Data are contained within the article and Supplementary Materials.

## Supplementary Materials

The following supporting information can be downloaded at: https://www.mdpi.com/article/10.3390/bios15050320/s1, Table S1: Summary of IL-6 sensing studies, detailing configuration, target, assay label, linear range, and limit of detection (LOD).
