# Supplementary material for: MIP-Modified Porous Silicon Optical Sensor for Interleukin-6 Label-Free Quantification
Source: Biosensors (Basel). 2025 May 17;15(5):320. doi: 10.3390/bios15050320 (PMC12109668; doi:10.3390/bios15050320)
Supplement: Supplementary file 1 [file biosensors-15-00320-s001.zip › biosensors-3589870-supplementary.pdf]

Supporting Information

Configuration	Target	Label	Linear range	LOD	Ref.
Colorimetric/solution-based	IL-6	Label free	3.3–125 µg/mL	1.59 µg/mL	[47]
Magnetic SERS sandwich immunoassay based on Au@Fe3O4 nanoring and Ag@4-MBA	IL-6	4-mercaptobenzoic acid (4-MBA)	0.1–1000 pg/mL	0.028 pg/mL	[52]
(P(o-PD))-based molecularly imprinted polymer (MIP) onto an oxygen-functionalized screen-printed carbon electrode with gold nanoparticles, 3-aminopropyltriethoxysilane (APTES), and glutaraldehyde (GA)	IL-6	Label-free	2-400 pg/mL	1.74 pg/mL	[49]
Molecular imprinted polymers (3-aminophenylboronic acid (APBA)) modified polycarbonate Microneedle array metalized with platinum and silver	IL-6	Label-free	1 pg/mL-10 ng/mL	1.74 pg/mL	[50]
Electropolymerized Methylene Blue and a Multi-Walled Carbon-Nanotube-Modified Screen-Printed Carbon Electrode	IL-6	Label free	0.001~1000.0 ng/mL	0.54 pg/mL	[51]
This work	IL-6	Label free	237-2370 ng/mL	300 ng/mL	

Table S1: Summary of IL-6 sensing studies, detailing configuration, target, assay label, linear range, and limit of detection (LOD).
